# Supplementary material for: Skin-related adverse events and their associated factors among Diabetic patients on insulin therapy
Source: PLoS One. 2026 Feb 2;21(2):e0320556. doi: 10.1371/journal.pone.0320556 (PMC12863474; doi:10.1371/journal.pone.0320556)
Supplement: Attachment — Submitted filename: Response to Reviewers.pdf [file pone.0320556.s006.pdf]

Dennis Patson Mbwambo
P.O.Box 6166
Dar Es Salaam
Tanzania
15th **October 2025**.

Editorial Manager
PLOS ONE

REF: Rebuttal to Review of the manuscript Titled "Skin-related adverse events and their associated factors among Diabetic patients on insulin therapy"

Dear editor,

I am writing to respectfully address the queries recommended following the assessment of the above-mentioned manuscript. There were eighteen issues: ten from the reviewer 3, and 8 from reviewer 5.All the issues have been responded accordingly.

Instruction No.1 (Reviwere-3)
Sampling interval: you now describe k = 2 systematic sampling (every other patient). A single sentence confirming that the first patient was chosen at random in each clinic session would satisfy the assumption of systematic sampling.

Rebuttal: Done, sentence has been revised to align with the above recommendation.

Documentation: Page 5

Instruction No.2 (Reviewer-3)
Power analysis: thank you for adding post hoc calculations. It would be clearer to move these numbers to the Limitations paragraph and simply note in Methods that post hoc power was estimated.

Rebuttal: Done. Numbers for post hoc have been moved from Method section to the limitations paragraph.

Documentation: Pages 9 & 18.

Instruction No.3 (Reviewer-3)
Table 1 now states three random BG readings define "controlled." Please add a short sentence explaining why this surrogate was preferred over fasting BG and how often readings were actually available (completeness)..

Rebuttal: Done. A sentence has been added to justify why RBG was used instead of Fasting Blood Glucose.

Documentation: Table 1.

Instruction No.4 (Reviewer-3)
Provide the list of covariates included in each multivariable model at the foot of Tables 5 and 6

Rebuttal: Done. Covariates included in each multivariable model has been listed at the foot of Tables 5 and 6

Documentation: Table 5 & 6.

Instruction No.5 (Reviewer-3)
Several confidence interval bounds do not include the point estimate or are reversed. This suggests residual transcription errors. E.g. Table 5: age group ≥ 45 y crude PR 0.51 with CI 1.03–1.14; sex crude PR 0.93 with CI 0.97–1.06; Table 6: age 25 44 crude PR 1.07 with CI 0.89–1.06.

Where crude PRs reverse direction after adjustment (e.g., insulin formulation), flag this in a sentence so readers understand the role of confounding.

Rebuttal: Done. We thank the reviewer for highlighting these points. The confidence interval errors noted were due to a data transfer error, which has now been corrected.

Documentation: Table 5 and 6.

Instruction No.6 (Reviewer-3)
The new comparisons with Ethiopia, India and Bangladesh are helpful. Where your prevalence is higher or lower, add a short clause on possible drivers (clinical training standards, availability of pens, sample age).

Rebuttal: Done.

Documentation: Paragraph 4 & 5 at discussion part.

Instruction No.7 (Reviewer-3)
The paragraph on insulin pen "protective advantage" still reads causal. Please rephrase so that the finding is framed as an observed association in your dataset. You may note that similar associations from Saudi Arabia and Lebanon increase the plausibility of a causal link—but, unless those papers explicitly demonstrate causality (I have not verified this), they should be cited only as supportive observational evidence. At the same time, acknowledge that residual confounding (for example, socioeconomic status or prior injection technique training) might partly explain the pattern. If robust longitudinal or interventional studies do show a causal benefit of pen devices, consider citing them; otherwise, emphasize that prospective confirmation is still needed.

Rebuttal: Done. The sentence has been rephrased

Documentation: Paragraph 8, page 18 at discussion part.

Instruction No.8 (Reviewer-3)
Discussion still contain causal verbs ("resulted in", "led to", "confer a protective advantage"). Because the study is cross sectional, please replace with associative phrasing ("was associated with", "showed lower prevalence") or explicitly frame any causal interpretation as speculative.

Rebuttal: Done. The sentence has been rephrased

Documentation: Paragraph 4, page 17 at discussion part.

Instruction No.9 (Reviewer-3)
Remove the repeated WHO pharmacovigilance reference (#10 and #16 appear identical)

Rebuttal: Done. Repeated has been removed

Documentation: References list.

Instruction No.9 (Reviewer-3)
Remove the repeated WHO pharmacovigilance reference (#10 and #16 appear identical)

Rebuttal: Done. Repeated has been resolved.

Documentation: References list.

Instruction No.10 (Reviewer-3)
Typographic clean up: "Forexample" → "For example"; double periods after citations (#8., #9…) to single period; etc

Rebuttal: Typographic clean-up has been done

Documentation: Through the document.

Instruction No.1 (Reviewer-5)
Conclusion section of a manuscript ought to summarise what was found in the study without repeating any figures/estimates. In this manuscript, some sentences in the conclusion section seem to belong to recommendations section rather than conclusion section.

Rebuttal: The recommendation statement has been removed from the conclusion and captured in the recommendation section.

Documentation: Conclusion and recommendation sections.

Instruction No.2 (Reviewer-5)
Some of the findings in the abstract's results' sub-section have their quantitative estimates omitted, and hence losing the meaning of the findings presentation.

e.g. why were the positive associated factors for scars formation not accompanied by their statistical estimates vivid in the multivariable section of the results section in the main text?

Rebuttal: Done. The abstract has been rephrased to capture all the relevant findings that were missing in previous version.

Documentation: Abstract section

Instruction No.3 (Reviewer-5)
It is important for the authors to be meticulous with the usage of technical words! At present, I had difficulties to believe if authors knew how to differentiate 'adverse events' from 'side effects' when it comes to pharmacological management of diabetes mellitus.

Reason: whereas diabetes keto-acidosis may be considered as an 'adverse event' among people with diabetes mellitus consuming oral hypoglycaemics (refer to the example provided by FDA adverse event reporting), the same 'diabetes keto-acidosis' is customarily regarded as a 'side effect' of insulin treatment among people with diabetes mellitus.

Recommendations: Authors need to consider another 'adverse event(s)' reported in literature that specifically addresses insulin usage among diabetics when building their argument about '***why the study was done***'

Rebuttal: The reviewer is correct that diabetic ketoacidosis (DKA) can be a complex case; it is a known consequence of insulin omission or failure (a treatment failure adverse event) and can also be a direct consequence of certain pharmacological agents like SGLT2 inhibitors.

Our specific focus, however, was on localized, injection-site complications such as abscesses and lipodystrophy. These are unequivocally classified as adverse events related to the method of drug administration, not the pharmacological action of insulin itself. They are a critical, yet often underreported, component of insulin safety profiles. Therefore, our decision to investigate the "prevalence and factors associated with skin-related adverse events among patients on insulin therapy" was a deliberate and justified one, stemming directly from a pharmacovigilance perspective aimed at improving patient safety and product use in real-world settings. This focus aligns with the core objective of pharmacovigilance: to detect, assess, understand, and prevent adverse effects or any other drug-related problem.

Instruction No.4 (Reviewer-5)
Can we confidently justify this study to had been a CROSS-SECTIONAL by design during the stipulated timeline?

Reason: Based on what authors have provided in the manuscript's methods section, there are clear indications that the study was RETROSPECTIVE by design on a de-facto basis!!!

Rebuttal:

We thank the reviewer for this insightful comment and the opportunity to clarify the study design. We understand the confusion that arose from the original phrasing in the methodology section and acknowledge that the language used could be misconstrued as indicating a retrospective design.

We wish to state unequivocally that the study was conceived, designed, and executed as a cross-sectional study. The core objective was to assess the point prevalence of injection site adverse events and associated factors at a single point in time. The following key aspects confirm the cross-sectional nature of our design:

Synchronous Assessment of Exposure and Outcome: All primary data on the outcome (presence of abscesses and scars) and key exposures (injection awareness, technique) were collected concurrently during a single study visit. The physical examination provided an objective, current assessment of adverse events, while the interview captured current awareness and practices. This synchronous measurement is the hallmark of a cross-sectional study.

The Use of Historical RBG Data as a Baseline Characteristic: The extraction of Random Blood Glucose (RBG) values from the preceding three months was not for a longitudinal or retrospective analysis of glycemic trends. Instead, it was solely to establish a baseline characteristic—the patient's approximate glycemic control status (controlled vs. uncontrolled)—at the time of the cross-sectional assessment. This is analogous to using the most recent blood pressure reading or weight recorded in a patient's file to characterize their current clinical status upon enrollment. No follow-up or retrospective tracking of patient outcomes over time was performed.

Recruitment and Single Data Collection Point: Participants were recruited and all study procedures (interview, physical exam, data extraction) were completed during a dedicated encounter. There was no follow-up period, and no analysis was based on the progression of variables over time.

The perceived "retrospective" element stemmed entirely from the description of the RBG data source. We recognize that our original wording was ambiguous and could imply a retrospective cohort design. We have therefore thoroughly refined the 'Data Collection Procedure' section in the revised manuscript to eliminate this confusion.

We are confident that with these clarifications, the manuscript now accurately and unambiguously reflects the cross-sectional design of our study. Thank you again for prompting this important clarification.

Documentation: Data collection-procedure sub section of the Materials and Methods section.

Instruction No.5 (Reviewer-5)

The description of how variables were treated before and during data analysis is missing in the manuscript

e.g. why was the variable 'age of participants' categorized? And maybe more interesting, what was the clinical/logistical basis of the cut-off age groups? (refer to table 2 in the results section for the logic behind the query!)

Rebuttal:

We thank the reviewer for this valuable comment. We agree that providing a clear rationale for the treatment of variables is essential for methodological transparency. The following explanations address the specific query regarding the categorization of age and our overall approach.

1. Rationale for Categorizing Continuous Variables (e.g., Age):

Continuous variables like age were categorized for both clinical and statistical reasons, which is a common and recommended practice in epidemiological studies.

Clinical and Behavioral Relevance: The risk of injection site complications is likely influenced by factors that vary across broad life stages rather than changing linearly with each year of age. Categorizing age allows us to capture these distinct clinical and behavioral patterns:

Young Adults (0-24 years): This group may include patients with Type 1 diabetes, who often start insulin at a young age. Their injection practices may be influenced by factors like transitioning from parental care to self-management, peer influence, and body image concerns, which could impact site rotation and hygiene.

Middle-Aged Adults (25-44 years): This is a period of complex life demands (career, family). Adherence to complex regimens, including proper injection technique, may compete with other responsibilities. This group may also have a longer duration of diabetes, leading to cumulative tissue damage.

Older Adults (45+ years): This population may face age-related challenges such as visual impairment, reduced dexterity, cognitive decline, and the presence of comorbidities. These factors can significantly hinder the ability to correctly administer insulin, leading to lipohypertrophy, abscesses, and scars.

Analytical Utility: Categorization facilitates a more intuitive and clinically meaningful interpretation of the results. It allows for easier comparison of risk profiles between distinct patient groups using odds ratios in logistic regression models, making the findings more actionable for clinicians.

2. Justification for the Specific Age Cut-offs (0-24, 25-44, 45+):

The selected cut-off points were not arbitrary. They were chosen based on **established life-stage and gerontological classifications** commonly used in public health and clinical research to reflect periods of shared physiological and lifestyle characteristics.

The grouping aligns with standards used by organizations like the World Health Organization and in numerous demographic and health surveys, which often define "young adulthood" as up to 24, "middle adulthood" as 25-44 or 25-59, and "older adulthood" as beginning at 45 or 50.

3. Action Taken in the Revised Manuscript:

In response to this comment, we have added the text to the Data Analysis section of the manuscript to enhance clarity:

We believe this clarification strengthens the methodological rigor of our work and thank the reviewer for prompting this important improvement.

Documentation: Data analysis section

Instruction No.6 (Reviewer-5)
I am still doubtful if the usage of 'modified poisson regression' was appropriate in assessing factors associated with both abscess and scar formation in this study findings.

Evidence: By default, poisson regression (and its modified versions) are reserved for otherwise 'rare events'. The fact that in this study they have reported the prevalence of scars formation to have been 46.7% and that of abscess as 22.2% rule-out the possibility for the data to fit in poisson regression model!

Reason: can one justify a prevalence rate of 46.7% to be a rare event?

Recommendations: Authors need to consult a 'chartered statistician' or a 'qualified biostatistician' for assistance into the matter. Likewise, in their subsequent re-submission, authors ought to make effort to attach their 'minimalised dataset' as well for reviewers' verification(s).

Rebuttal:

We thank the reviewer for raising this important methodological point. We understand the concern, which stems from a common misconception. However, the use of **modified Poisson regression with robust error variances** is not only appropriate but is, in fact, a widely recommended and statistically sound method for analyzing cross-sectional data with binary outcomes and high prevalence, precisely to avoid the limitations of logistic regression in such scenarios.

The reviewer is correct that standard Poisson regression is typically used for count data and can be sensitive to over-dispersion. However, the "modified" version we employed, as pioneered by Zou (2004) and others, is a specific adaptation for binary outcomes to directly estimate Risk Ratios (**RR**) or Prevalence Ratios (PR).

Given that our study had a cross-sectional design and reported a high-prevalence outcome (scars at 46.7%), using logistic regression would have been less appropriate, as it would have produced inflated Odds Ratios. Our choice of **modified Poisson regression was a deliberate and statistically rigorous decision to provide the most accurate and interpretable measure of association (the Prevalence Ratio).**

We have ensured that this analysis was conducted under the guidance of a qualified biostatistician. The methodology is sound, and the results presented in Tables 5 & 6 are a valid representation of the factors associated with injection site adverse events.

Regarding the request for a minimal dataset, we will be happy to provide a fully anonymized dataset as part of the supplementary materials upon manuscript acceptance, in accordance with the journal's data-sharing policy.

Documentation: A statement justifying the rationale for using the modified Poisson regression has been capture at data analysis section.

Instruction No.7 (Reviewer-5)
Why do some estimates reported to be outside the written 95% confidence interval?

Evidence: refer to age group ≥ 45 years aPR and its corresponding 95% C.I. in table 5 (aPR=0.6, 95% C.I.: 0.99 – 1.15) !!!

Note: it is highly probable that there was profound model misspecification and therefore entire thinking of fitting a POISSON REGRESSION model was invalid in this study!

Recommendations: Consult a '**chartered statistician**' or a **qualified biostatistician** for urgent assistance on the matter.

Rebuttal:

We thank the reviewer for their exceptionally astute observation and for identifying this critical inconsistency in Table 5. The reviewer is absolutely correct: a point estimate (aPR=0.60) cannot logically exist outside its own 95% confidence interval (0.99 – 1.15). This is a statistical impossibility that indicates an error in the preparation of the table or the underlying analysis, and we sincerely apologize for this oversight.

Upon immediate re-investigation of our statistical output, we have identified the source of the error. The value of 0.60 was the crude p-value from the univariate analysis for that variable, which was mistakenly transcribed into the column for the Adjusted Prevalence Ratio (aPR) in Table 5. The correct aPR for the ≥45 years age group is **1.06**, and the corresponding **95% CI is 0.99 – 1.15**. This correction resolves the logical contradiction and shows a non-significant association, which is consistent with the confidence interval containing the null value of 1.0.

Actions Taken and Reassurance on Methodology:

Full Data Audit: We have conducted a full audit of the data in **Tables 5 and 6** against the original output to correct any other transcription errors. This has ensured that all point estimates and their confidence intervals are now consistent and accurately reported in the revised manuscript.

Reaffirmation of Model Validity: This error was a result of manual data handling during table creation, not a flaw in the statistical model itself. We reconfirm that the modified Poisson

regression model was correctly specified and appropriate for our study objectives to estimate Prevalence Ratios for our common binary outcomes, as defended in our previous response.
We deeply regret this error and are grateful for the reviewer's meticulous scrutiny, which has been instrumental in improving the accuracy and integrity of our work. The revised manuscript will contain the corrected tables, and we are confident that these corrections strengthen the validity of our reported findings.

Documentation: Table 5.

Instruction No.8 (Reviewer-5)
Authors are advised to stick to 'standards of scientific writing' in their manuscript.

Reason: interpretation of findings is customarily reserved to discussion section of the manuscript. It was surprising to see interpretation of findings made in the results section of this manuscript!

Evidence: Furthermore, those employing insulin pens exhibited a prevalence 0.13 times lower when compared to individuals utilising vials and syringes (aPR = 0.13; 95%CI: 0.04-0.48; p = 0.002)

Rebuttal: Done. The interpretation of findings has been removed from the Results section and is now appropriately placed in the Discussion. The manuscript has been revised to adhere to standards of scientific writing.

Documentation: Results and discussion sections of the manuscript

Request for Reconsideration

I kindly ask that you reevaluate the revised manuscript in light of this clarified information. I am open to further discussion or revisions to resolve any remaining concerns. Thank you for your time and attention to this matter.
Please contact me at +255658333469/dennis.mbwambo@gmail.com with any questions.
Sincerely,

Denis Patson Mbwambo
The auther.
