# Supplementary material for: Skin-related adverse events and their associated factors among Diabetic patients on insulin therapy
Source: PLoS One. 2026 Feb 2;21(2):e0320556. doi: 10.1371/journal.pone.0320556 (PMC12863474; doi:10.1371/journal.pone.0320556)
Supplement: Attachment — Submitted filename: Review of Article-20250414.docx [file pone.0320556.s001.docx]

**Summary:**

This hospital-based analytical cross-sectional study investigated injection-site complications among 428 diabetic patients on insulin therapy in Dar es Salaam, Tanzania, from February to May 2024 with the prevalence of abscess and scar formation was 22.2% and 46.7%, respectively. In addition, some key factors were found to associated with abscess included improper injection technique, lack of site rotation, and reduced risk with insulin pen use. And scar formation was proved to link with poor site rotation, uncontrolled blood glucose, and using insulin or syringes from community drug outlets. Thus, these findings highlight the need for regular patient education on proper insulin injection practices to reduce complications and improve treatment adherence. However, there are still some comments that needed to be addressed by the author.

**Comments:**

1. The Inconsistent number: the author mentions 530 million people with DM in 2019 but later refer to 463 million. These figures should be consistent or clarified (perhaps 530 million is a more recent estimate?).
2. When the author mentions, “in recent years,” or “just in two decades,” it is better to clarify the starting point if possible (e.g., “since 2000”).
3. "Cross-section" → should be "cross-sectional". And "analytical cross-section study" → "analytical cross-sectional study".
4. "Three-month random blood glucose readings" → "random" may be confusing in this context; better to clarify. For example, "random blood glucose values recorded over the past three months".
5. The phrase “we recruited one eligible subject after skipping another” is vague. Better phrasing: “Systematic sampling was used with a predetermined interval (every second eligible patient) until the required sample size per facility was reached.”
6. Redundant and Disorganized Presentation: Some key findings (e.g., syringe reuse and insulin source) are repeated across multiple paragraphs. The flow between patient characteristics, prevalence data, and factors associated with outcomes lacks structure, which may confuse readers and dilute key messages.
7. Language and Formatting Issues: There are several grammatical errors, awkward phrases (“per a needle”), and inconsistent formatting of numbers and percentages (e.g., missing spaces or switching between formats). These stylistic issues reduce clarity and compromise the scientific tone of the section.
