# Supplementary material for: Skin-related adverse events and their associated factors among Diabetic patients on insulin therapy
Source: PLoS One. 2026 Feb 2;21(2):e0320556. doi: 10.1371/journal.pone.0320556 (PMC12863474; doi:10.1371/journal.pone.0320556)
Supplement: Attachment — Submitted filename: Response to Reviewers.docx [file pone.0320556.s003.docx]

Dennis Patson Mbwambo
P.O.Box 6166
Dar Es Salaam
Tanzania
**26^th^ May 2025**.

Ronalyn M. Ramos
PLOS ONE

**REF:** **Rebuttal to Review of the manuscript Titled “Skin-related adverse events and their determinants among Diabetic patients on insulin therapy in Tanzania”**

Dear Dr. Misbah,

I am writing to respectfully address the queries recommended following the assessment of the above-mentioned manuscript. There were two issues and each has been responded accordingly.

1. Thank you for updating your data availability statement. You note that your data are available within the Supporting Information files, but no such files have been included with your submission. At this time we ask that you please upload your minimal data set as a Supporting Information file, or to a public repository such as Figshare or Dryad.

**Rebuttal**: Data availability statement has been rectified.

**Supporting Documentation**

I have attached the following for your review:

- A revised manuscript without mark-up

**Request for Reconsideration**

I kindly ask that you reevaluate the revised manuscript in light of this clarified information. I am open to further discussion or revisions to resolve any remaining concerns.

Thank you for your time and attention to this matter. Please contact me at +255658333469/dennis.mbwambo@gmail.com with any questions.

Sincerely,
Denis Patson Mbwambo
The auther.
