# Supplementary material for: Skin-related adverse events and their associated factors among Diabetic patients on insulin therapy
Source: PLoS One. 2026 Feb 2;21(2):e0320556. doi: 10.1371/journal.pone.0320556 (PMC12863474; doi:10.1371/journal.pone.0320556)
Supplement: Attachment — Submitted filename: Response_to_Reviewers_auresp_2.docx [file pone.0320556.s005.docx]

Dennis Patson Mbwambo
P.O.Box 6166
Dar Es Salaam
Tanzania
**25^th^ July 2025**.

Editorial Manager
PLOS ONE

**REF:** **Rebuttal to Review of the manuscript Titled “Skin-related adverse events and their associated factors among Diabetic patients on insulin therapy”**

Dear editor,

I am writing to respectfully address the queries recommended following the assessment of the above-mentioned manuscript. There were twenty six issues from all the two reviewers each has been responded accordingly.

**Instruction No.1**
Please ensure that you refer to Table 1 in your text as, if accepted, production will need this reference to link the reader to the Table.

**Rebuttal**: Done, Table 1 has been referred to in the text.

**Documentation**: Page 6

**Instruction No.2**
Please note that funding information should not appear in the Acknowledgments section/Funding section or Any other areas of your Manuscript. We will only publish funding information present in the Funding Statement section of the online submission form. Please remove any funding-related text from the manuscript

**Rebuttal**: Done. Funding information has been removed from the manuscript.

**Documentation:** Page 20.

**Instruction No.3**
Please amend the title either on the online submission form or in your so that they are identical.

**Rebuttal**: Done. The title has been amended in the online submission form and cover letter. The tittle in online submission is now identical to that on the manuscript.

**Documentation:** Online submission form, Cover letter and the manuscript tittles.

**Request for Reconsideration**

I kindly ask that you reevaluate the revised manuscript in light of this clarified information. I am open to further discussion or revisions to resolve any remaining concerns.

Thank you for your time and attention to this matter. Please contact me at +255658333469/dennis.mbwambo@gmail.com with any questions.

Sincerely,
Denis Patson Mbwambo
The auther.
