# Supplementary material for: Skin-related adverse events and their associated factors among Diabetic patients on insulin therapy
Source: PLoS One. 2026 Feb 2;21(2):e0320556. doi: 10.1371/journal.pone.0320556 (PMC12863474; doi:10.1371/journal.pone.0320556)
Supplement: Attachment — Submitted filename: REVIEWERS queries - PONE-D-25-08926R2.doc [file pone.0320556.s004.doc]

**REVIEWERS’ QUERIES/COMMENTS/RECOMMENDATIONS FOR PONE-D-25-08926R2**.

**GENERAL COMMENTS/QUERIES/RECOMMENDATIONS**:

1. The manuscript describes important clinical phenomena to all practicing endocrinologists/physicians the world over. If done

correctly, it would contribute to otherwise unknown quantitative estimates of observed clinical phenomena in African settings.

2. There are several flawed grammatical, syntactic, technical as well as logical attributes throughout the document as shown in the specific comments the underneath.

e.g. since the entire study had its **gravity** on ‘*skin adverse events*’, that are by default *unfavourable and rare events*, and almost always sporadic (rather than prevalent!), didn’t investigators/authors consider the need for the study design to have been ‘*prospective observational clinical research*’ by design?

Otherwise, why doesn’t the manuscript have page numbers?

**SPECIFIC COMMENTS/QUERIES/RECOMMENDATIONS**:

| **Reviewers’ query/recommendations/comments** | **Section** |
| --- | --- |
| 1. Conclusion section of a manuscript ought to summarise what was found in the study without repeating any figures/estimates. In this manuscript, some sentences in the conclusion section seem to belong to **recommendations section** rather than conclusion section.   **Evidence:** “Consequently, we advocate for longitudinal or controlled studies to ascertain causality”. | Abstract |
| 2. Some of the findings in the abstract’s results’ sub-section have their quantitative estimates omitted, and hence losing the meaning of the findings presentation.   e.g. why were the positive associated factors for scars formation not accompanied by their statistical estimates vivid in the multivariable section of the results section in the main text?  **Note**: in most cases, it is the abstract section that will ever be read in future, once the article becomes published. As a summary of the entire manuscript, it still needs to be as informative as possible! | Abstract |

| **Reviewers’ query/recommendations/comments** | **Section** |
| --- | --- |
| 3. It is important for the authors to be meticulous with the usage of technical words! At present, I had difficulties to believe if authors knew how to differentiate ‘*adverse events*’ from ‘*side effects*’ when it comes to pharmacological management of diabetes mellitus.     **Reason:** whereas diabetes keto-acidosis may be considered as an ‘*adverse event*’ among people with diabetes mellitus consuming oral hypoglycaemics (refer to the example provided by FDA adverse event reporting), the same ‘diabetes keto-acidosis’ is customarily regarded as a ‘*side effect*’ of insulin treatment among people with diabetes mellitus.  **Recommendations:** Authors need to consider another ‘adverse event(s)’ reported in literature that specifically addresses insulin usage among diabetics when building their argument about ‘***why the study was done***’ | Introduction |
| 4. Can we confidently justify this study to had been a CROSS-SECTIONAL by design during the stipulated timeline?   **Reason:** Based on what authors have provided in the manuscript’s methods section, there are clear indications that the study was RETROSPECTIVE by design on a *de-facto* basis!!!  **Evidence:**  “Data were meticulously gathered from both patient files and direct interviews. The data extracted from patient records encompassed random blood glucose readings, sociodemographic details, and other pertinent information.”  (refer to 2^nd^ paragraph in the sub-section **data collection procedure**)  And again  “In addition to the physical examination for the identification of existing abscesses and scars, patients were queried about the development of any abscesses or scars within the twelve months preceding the interview.”  **Recommendations:** I would kindly request authors to either consult **a clinical epidemiologist** or simply refer to **any basic epidemiology textbook** that may provide a description for the differences between the two study designs. | Methods |

| **Reviewers’ query/recommendations/comments** | **Section** |
| --- | --- |
| 5. The description of how variables were treated before and during data analysis is missing in the manuscript   e.g. why was the variable ‘age of participants’ categorized? And maybe more interesting, what was the clinical/logistical basis of the cut-off age groups? (refer to table 2 in the results section for the logic behind the query!)  **Reason:** Do authors understand that categorizing an otherwise naturally occurring continuous variable during data analysis is associated with some loss of information? That in turn may render the variable ‘not significant’ (type II error), especially if the study was under-powered?? | Methods |
| 6. I am still doubtful if the usage of ‘modified poisson regression’ was appropriate in assessing factors associated with both abscess and scar formation in this study findings.   **Evidence:** By default, poisson regression (and its modified versions) are reserved for otherwise ‘rare events’. The fact that in this study they have reported the prevalence of scars formation to have been 46.7% and that of abscess as 22.2% rule-out the possibility for the data to fit in poisson regression model!  **Reason:** can one justify a prevalence rate of 46.7% to be a rare event?  **Recommendations:** Authors need to consult a ‘chartered statistician’ or a ‘qualified biostatistician’ for assistance into the matter. Likewise, in their subsequent re-submission, authors ought to make effort to attach their ‘minimalised dataset’ as well for reviewers’ verification(s). | Results |
| 7. Why do some estimates reported to be outside the written 95% confidence interval?   **Evidence:** refer to age group ≥ 45 years aPR and its corresponding 95% C.I. in table 5 (aPR=0.6, 95% C.I.: 0.99 – 1.15) !!!  **Note:** it is highly probable that there was profound model misspecification and therefore entire thinking of fitting a POISSON REGRESSION model was invalid in this study!  **Recommendations:** Consult a ‘**chartered statistician**’ or a **qualified biostatistician** for urgent assistance on the matter. | Results |

| **Reviewers’ query/recommendations/comments** | **Section** |
| --- | --- |
| 8. Authors are advised to stick to ‘standards of scientific writing’ in their manuscript.   **Reason:** interpretation of findings is customarily reserved to discussion section of the manuscript. It was surprising to see interpretation of findings made in the results section of this manuscript!  **Evidence:** Furthermore, those employing insulin pens exhibited a prevalence 0.13 times lower when compared to  individuals utilising vials and syringes (aPR = 0.13; 95%CI: 0.04-0.48; p = 0.002) | Results |
| 9. There is evidence of vivid erroneous interpretation of analysed findings in this manuscript!   e.g. Furthermore, those employing insulin pens exhibited a prevalence 0.13 times lower when compared to individuals utilising vials and syringes (aPR = 0.13; 95%CI: 0.04-0.48; p = 0.002)  **Why it was wrong?** – when reporting how much lower for cases where the estimates are below a unit, the proper interpretation is to consider the difference from the unit  **Recommendations:** should authors consider to report the way they have reported (highly recommended for that to appear in the DISCUSSION section and not where it has been cited in text!) then, they ought to write  “ ..exhibited a a prevalence of **87** times lower when compared to…”  **Reason:** the true **relative** lower value being = 100-13. | Results |
